# Supplementary material for: Machine learning suggests polygenic risk for cognitive dysfunction in amyotrophic lateral sclerosis
Source: EMBO Mol Med. 2020 Dec 3;13(1):e12595. doi: 10.15252/emmm.202012595 (PMC7799365; doi:10.15252/emmm.202012595)

APPENDIX

Table of Contents:

CReATe Consortium Author List …………………………………………………………..2

Supplementary Figures ……………………………………………………………………..5

Appendix Figure S1. Principal Components in the CReATe PGB cohort ……………5

Appendix Figure S2. *p* value calculation for sCCA modeling …………………………6

Appendix Figure S3. Variables selected in sCCA modeling excluding patients with primary lateral sclerosis and progressive muscular atrophy ….7

Appendix Figure S4. wPRS calculated with and without variants in high LD ………..8

Appendix Figure S5. Reduced cortical thickness in ALS patients relative to healthy controls …………………………………………………9

Appendix Figure S6. Magnitude of TDP-43 pathology in ALS cases at autopsy relative to wPRS ………………………………………………10

CReATe Consortium Author List:

**Name**	**Location**	**Role**	**Contribution**
Sumaira Hussain	University of Miami, USA	Project Manager	Regulatory lead; multi-center oversight and project management
Anne Cooley	University of Miami, USA	Associate Project & Data Manager	Multi-center oversight and project management; data query resolution
Yindi Li	University of Miami, USA	Statistical programmer	Data management
Marielle Wallace	University of Miami, USA	Data Manager	Data management
Julie Steele	University of Miami, USA	Outcomes Coordinator	Training and certification of site coordinators in outcome measures
Jessica P. Hernandez	University of Miami, USA	Research Coordinator	Visit coordination and data collection
Jessica Medina	University of Miami, USA	Research Coordinator	Visit coordination and data collection
Maria Elena Paredes	University of Miami, USA	Research Coordinator	Visit coordination and data collection
Kristina M Reyes	University of Miami	Research Coordinator	Visit coordination and data collection
Ashley Manso	University of Miami, USA	Research Coordinator	Data collection and review
Natalia Ravelo	University of Miami, USA	Research Coordinator	Visit coordination and data collection
Wendy Levy	University of Miami, USA	Research Coordinator	Visit coordination and data collection
Patrice Whitehead	John P Hussman Institute for Human Genomics	Biorepository Technical Director	CReATe Biorepository laboratory oversight
Stephan Zuchner, MD, PhD	University of Miami, USA	Co-investigator	CReATe Consortium Genetic Working Group member
Richard Barohn, MD	University of Kansas Medical Center, USA	Sub-investigator	Data collection
Mamatha Pasnoor, MD	University of Kansas Medical Center, USA	Sub-investigator	Data collection
Omar Jawdat, MD	University of Kansas Medical Center, USA	Sub-investigator	Data collection
Duaa Jabari, MD	University of Kansas Medical Center, USA	Sub-investigator	Data collection
Constantine Farmakidis, MD	University of Kansas Medical Center, USA	Sub-investigator	Data collection
Melanie Glenn, MD	University of Kansas Medical Center, USA	Sub-investigator	Data collection

Mazen M Dimachkie, MD	University of Kansas Medical Center, USA	Sub-investigator	Data collection
Laura Herbelin	University of Kansas Medical Center, USA	Site Lead Coordinator	Visit coordination and data collection
Hellen Tanui	University of Kansas Medical Center, USA	Research Coordinator	Visit coordination and data collection
Sherri Anderson	University of Kansas Medical Center, USA	Research Coordinator	Visit coordination and data collection
Michaela Walker	University of Kansas Medical Center, USA	Research Coordinator	Visit coordination and data collection
Tina Liu	University of Kansas Medical Center, USA	Research Coordinator	Visit coordination and data collection
Ayla McCally	University of Kansas Medical Center, USA	Research Coordinator	Visit coordination and data collection
Andrew Heim	University of Kansas Medical Center, USA	Research Coordinator	Visit coordination and data collection
Melissa Currence	University of Kansas Medical Center, USA	Research Coordinator	Visit coordination and data collection
Yolanda Harness	University of Kansas Medical Center, USA	Research Coordinator	Visit coordination and data collection
Kathryn Johns	University of Kansas Medical Center, USA	Research Coordinator	Visit coordination and data collection
Amruta Joshi	University of Virginia, USA	Research Coordinator	Visit coordination and data collection
Mary Wagoner	University of Virginia, USA	Research Coordinator	Visit coordination and data collection
Stephanie Lowenhaupt	University of Virginia, USA	Research Coordinator	Visit coordination and data collection
Gil Gutierrez	University of California San Diego, USA	Research Coordinator	Visit coordination and data collection
Danielle Vaporean-Bussey	University of California San Diego, USA	Research Coordinator	Visit coordination and data collection
Rose Previte	University of California San Diego, USA	Research Coordinator	Visit coordination and data collection
Aaryn Greer-Belfer	University of California San Diego, USA	Research Coordinator	Visit coordination and data collection
Ivy Chippendale Cass	University of California San Diego, USA	Research Coordinator	Visit coordination and data collection
Sahana Malik	University of California San Diego, USA	Research Coordinator	Visit coordination and data collection
Jeri Sieren	University of Iowa, USA	Research Coordinator	Visit coordination and data collection
Emilee Gibson	University of Iowa, USA	Research Coordinator	Visit coordination and data collection

Amy Conger	UT Southwestern Medical Center, USA	Research Coordinator	Visit coordination and data collection
Nina Gorham	UT Southwestern Medical Center, USA	Research Coordinator	Visit coordination and data collection
Simon Carty	California Pacific Medical Center, USA	Research Coordinator	Visit coordination and data collection
Jennifer Milan	California Pacific Medical Center, USA	Research Coordinator	Visit coordination and data collection
Debbie Hastings	Cleveland Clinic, USA	Research Coordinator	Visit coordination and data collection
Irys Caristo	Cleveland Clinic, USA	Research Coordinator	Visit coordination and data collection
Pamella Kittrell	University of Texas Health Science Center at San Antonio, USA	Research Coordinator	Visit coordination and data collection
Mozhdeh Marandi	Wake Forest School of Medicine, USA	Research Coordinator	Visit coordination and data collection
Summer Dawn Harris	Wake Forest School of Medicine, USA	Research Coordinator	Visit coordination and data collection
Mitchell John Reddan	Stanford, USA	Research Coordinator	Visit coordination and data collection
Jacinda Sampson, MD	Stanford, USA	Sub-investigator	Data Collection
Neelam Goyal, MD	Stanford, USA	Sub-investigator	Data Collection
Cherie Martinson	Twin Cities, USA	Research Coordinator	Visit coordination and data collection
Radhika Edpuganti	Twin Cities, USA	Research Coordinator	Visit coordination and data collection
Ezgi Tiryaki, MD	Twin Cities, USA	Sub-investigator	Data Collection
Rebecca Schule, MD, PhD	University of Tubingen, Germany	Co-investigator	CReATe Consortium Genetic Working Group member
Marka Blittersvijk	Jacksonville, USA	Co-investigator	CReATe Consortium Genetic Working Group member

Supplementary Figures:

Appendix Figure S1. Principal Components in the CReATe PGB cohort. Scatterplot showing the first principal component plotted against the second principal component from a principal components analysis conducted in the CReATe PGB cohort.

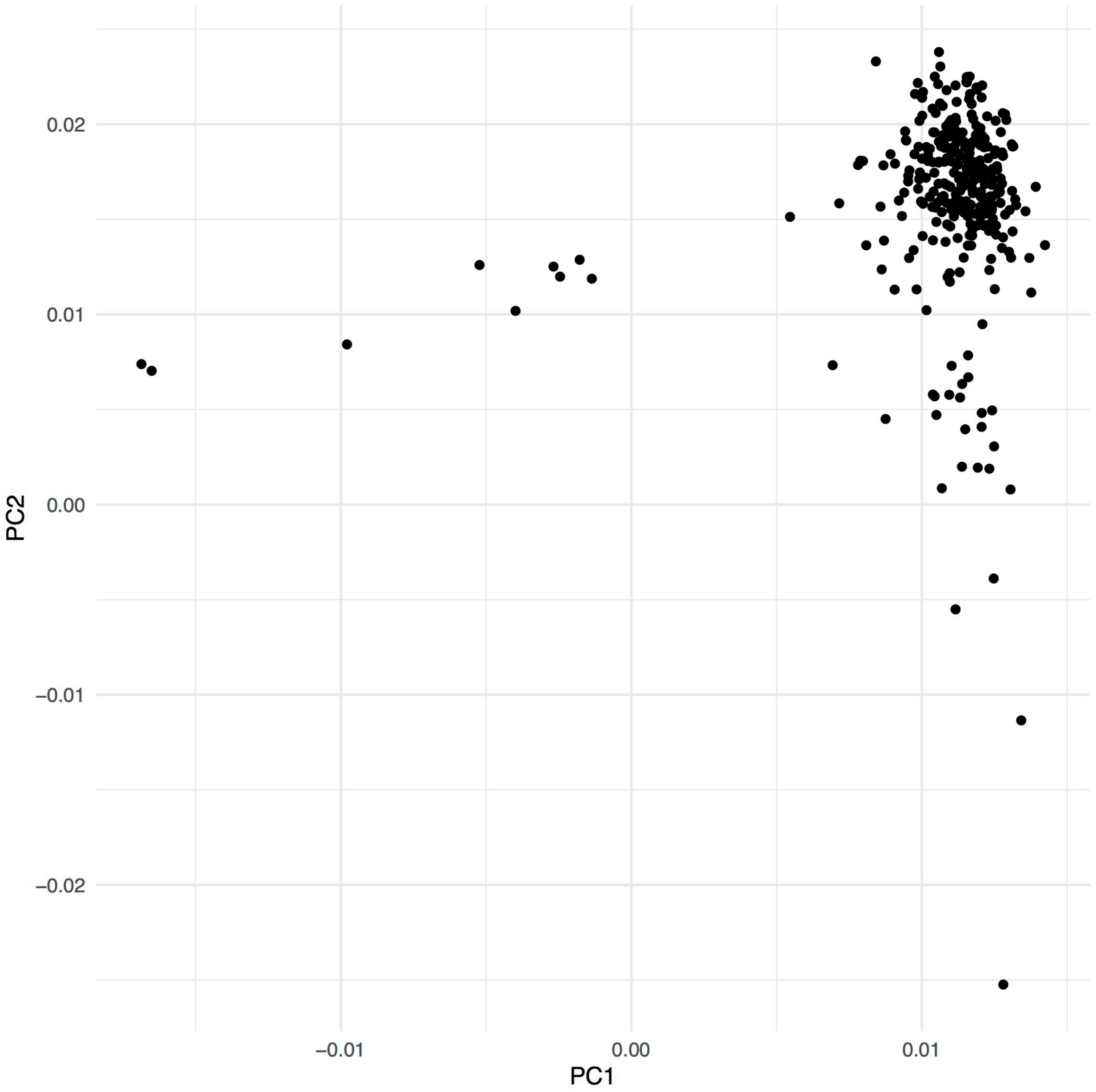

Appendix Figure S2. *p* value calculation for sCCA modeling. Histogram showing the frequency of canonical correlations achieved for sCCA modeling using 10,000 bootstrapped random permutations of the data. The vertical turquoise line denotes the median canonical correlation achieved under sCCA modeling of the true unpermuted data. The *p* value is the proportion of times the median canonical correlation (*R*) for sCCA modeling of the true, unpermuted data was achieved by sCCA modeling of the randomly permuted data.

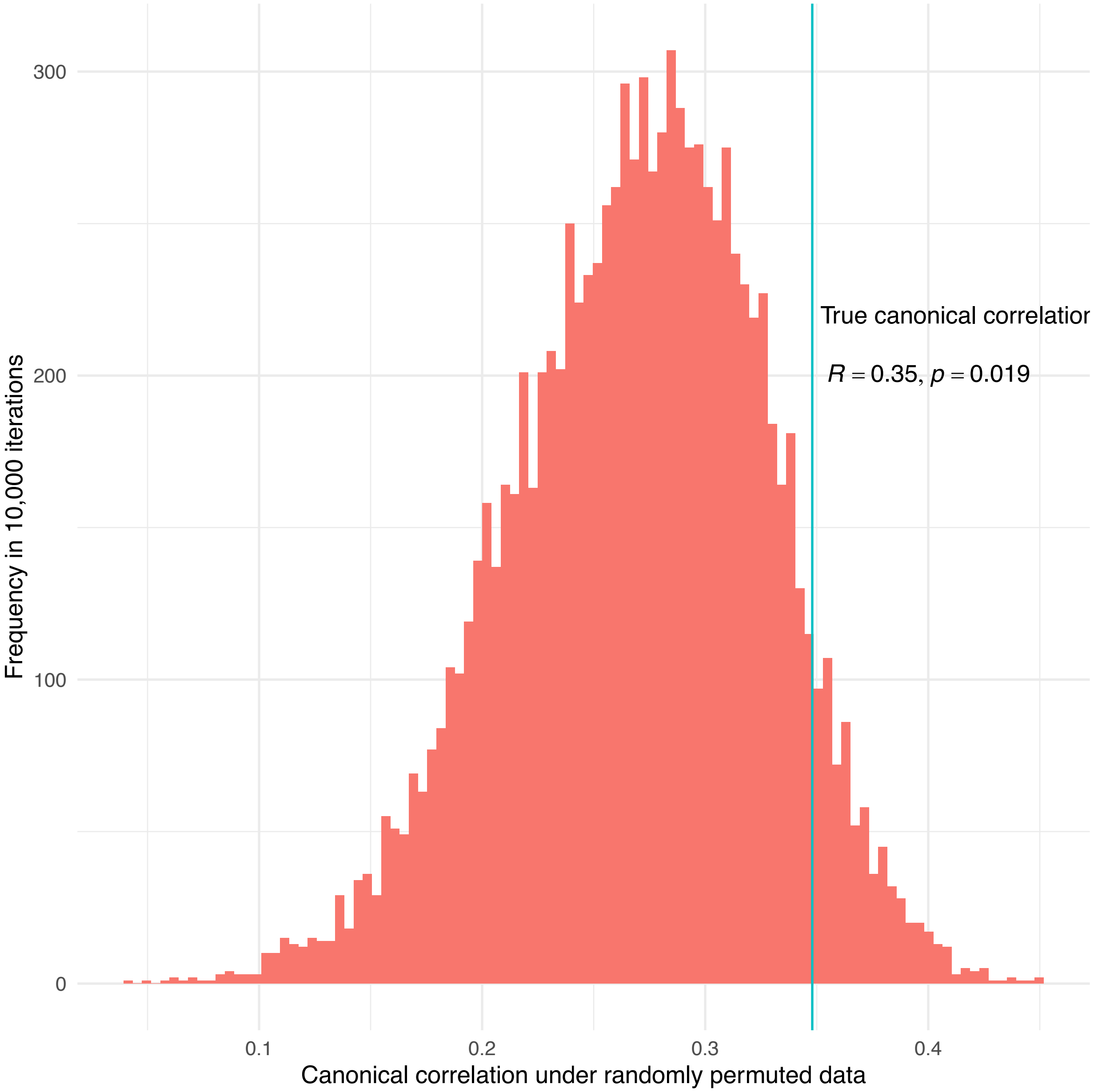

Appendix Figure S3. Variables selected in sCCA modeling excluding patients with primary lateral sclerosis and progressive muscular atrophy.

A) Bar graphs demonstrating the proportion of times out of 10,000 iterations that each of the 11 clinical variables were selected by sCCA under true modeling (turquoise) and modeling under the null hypothesis (coral).

B) Bar graphs demonstrating the number of times out of 10,000 randomly-bootstrapped sCCAs that each of the 45 SNPs were selected by sCCA under true modeling (turquoise) and modeling under the null hypothesis (coral). SNPs are organized according to prior genome-wide association with ALS or joint association with ALS and FTD.

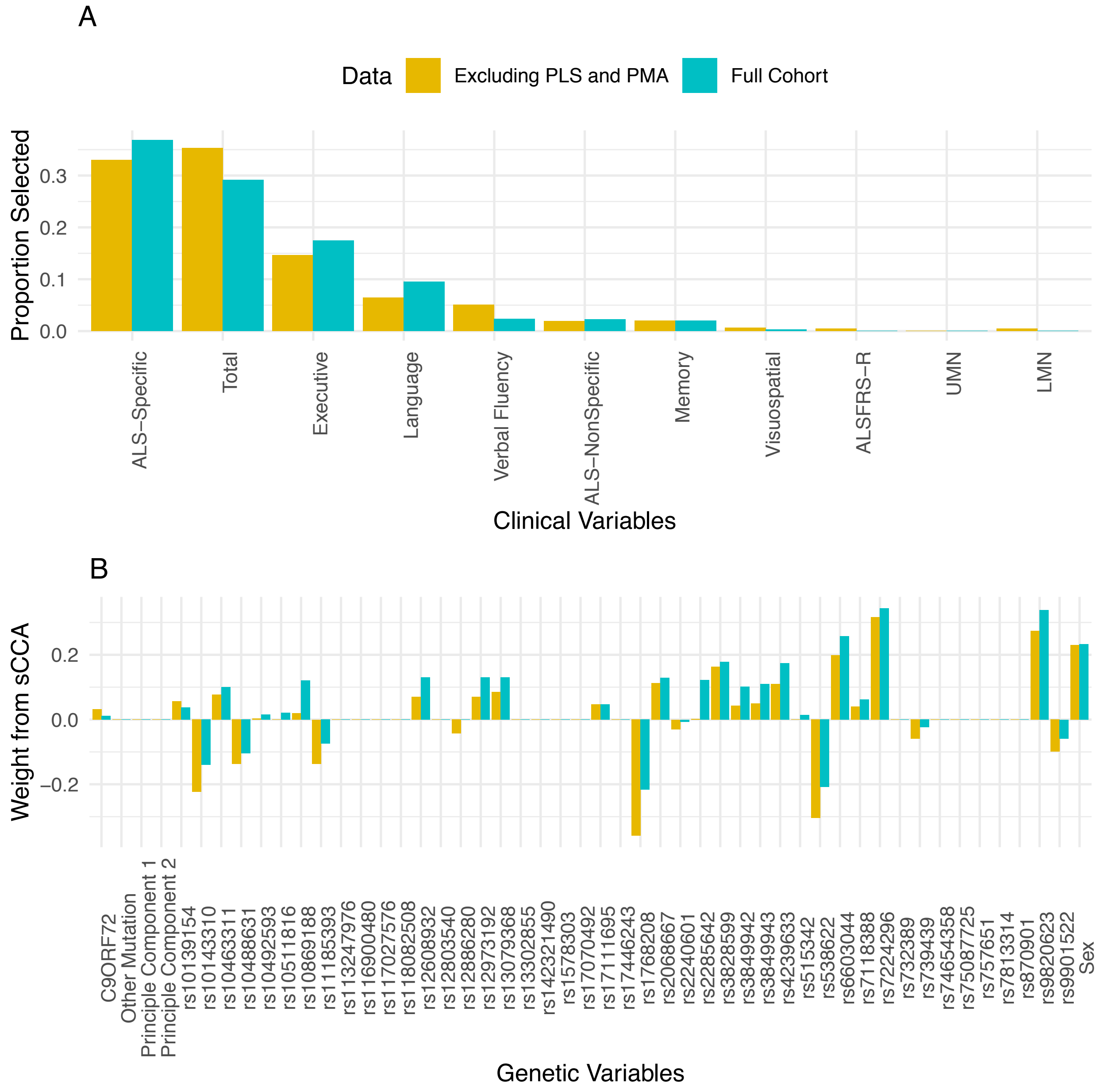

Appendix Figure S4. wPRS calculated with and without variants in high LD. Scatterplot depicting Pearson's correlation between the wPRS calculated using all genetic variables and the wPRS calculated excluding SNPs in high linkage disequilibrium.

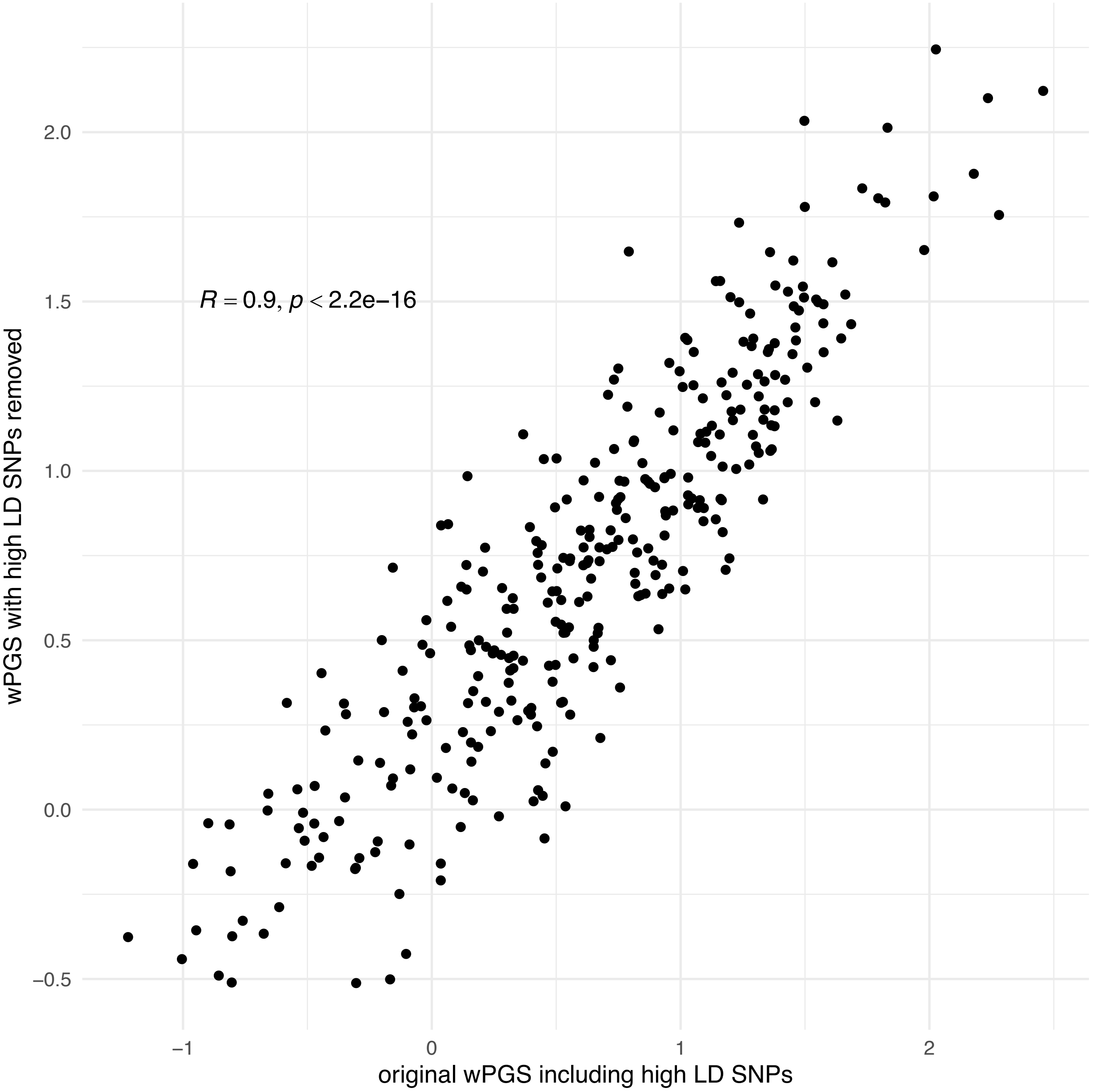

Appendix Figure S5. Reduced cortical thickness in ALS patients relative to healthy controls. ALS patients from the UPenn Biobank neuroimaging cohort displayed widespread cortical thinning relative to age, sex, and education-matched healthy controls in the frontal and temporal lobes. The heatmap indicates the associated T-statistic for each voxel, with light yellow representing the highest value.

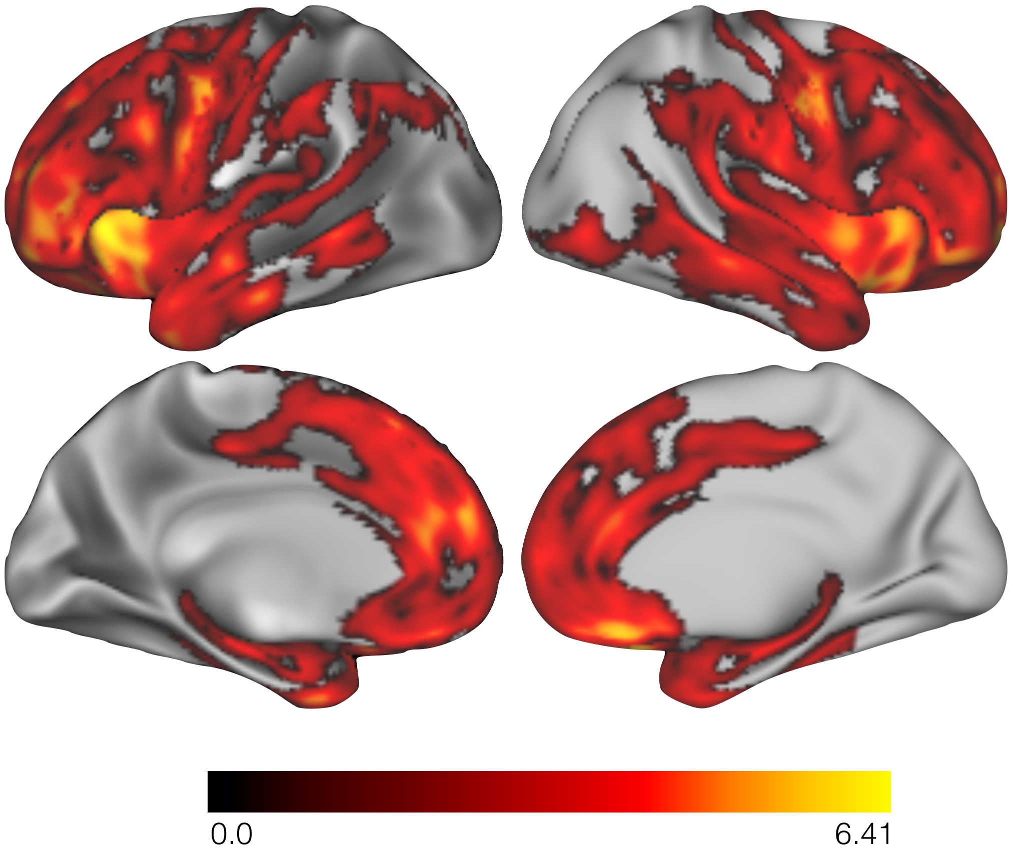

Appendix Figure S6. Magnitude of TDP-43 pathology in ALS cases at autopsy relative to wPRS. Beeswarm boxplots of ordinal measures of TDP-43 pathology in ALS cases the UPenn Biobank autopsy cohort relative to wPRS in the cingulate cortex, motor cortex, middle frontal cortex, superior / middle temporal cortex, and hippocampus. The central bands indicate the median, the box indicates the interquartile range (IQR) between the first and third quartiles, and the upper and lower whiskers indicate the largest or smallest value no further than the IQR multiplied by 1.5. For each brain region each data point represents a unique case.

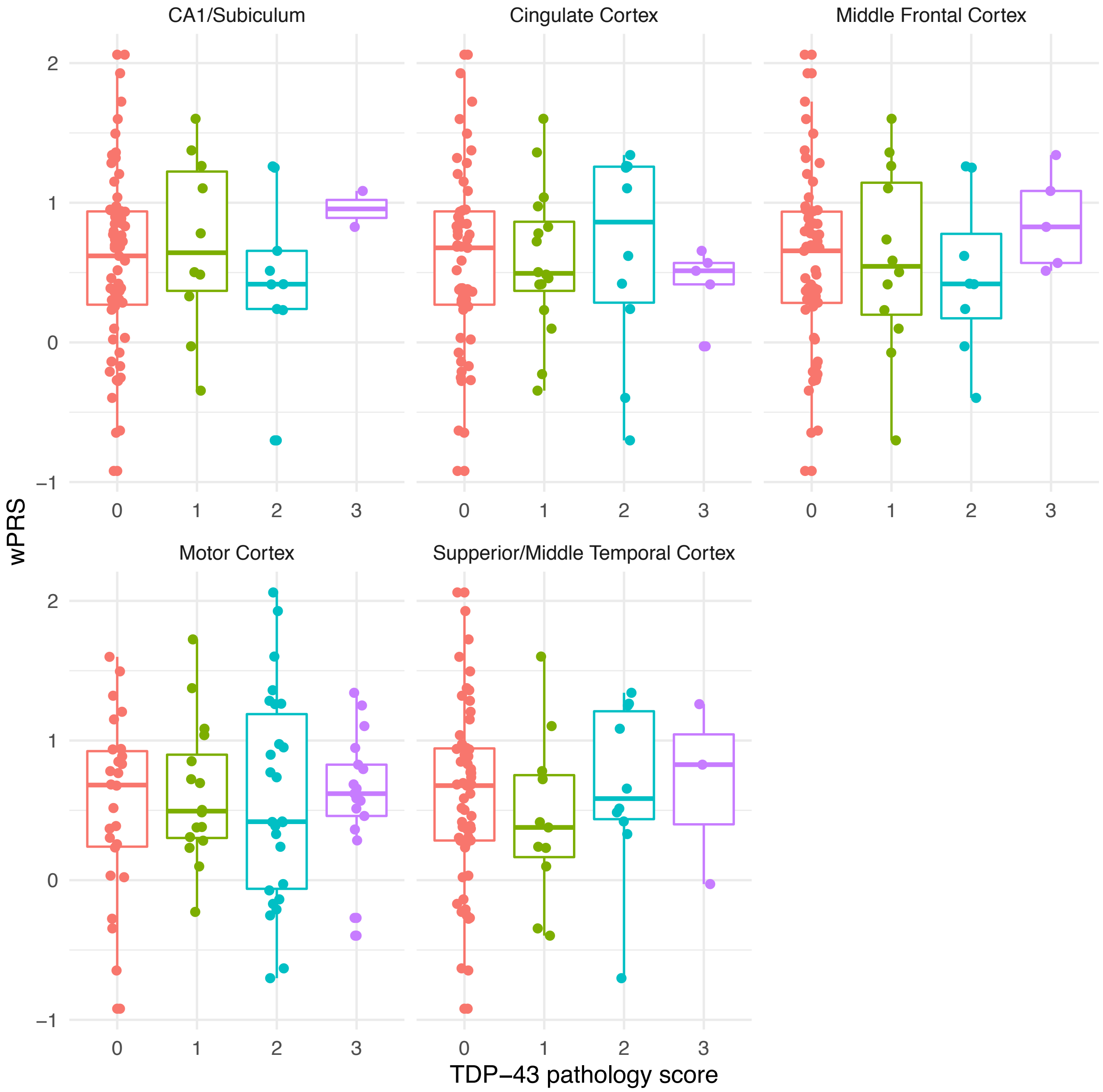

Supplement: Supplementary file 1 — Appendix [file EMMM-13-e12595-s001.pdf]
