# Supplementary material for: Machine learning suggests polygenic risk for cognitive dysfunction in amyotrophic lateral sclerosis
Source: EMBO Mol Med. 2020 Dec 3;13(1):e12595. doi: 10.15252/emmm.202012595 (PMC7799365; doi:10.15252/emmm.202012595)
Supplement: Supplementary file 2 — Expanded View Figures PDF [file EMMM-13-e12595-s002.pdf]

Expanded View Figures

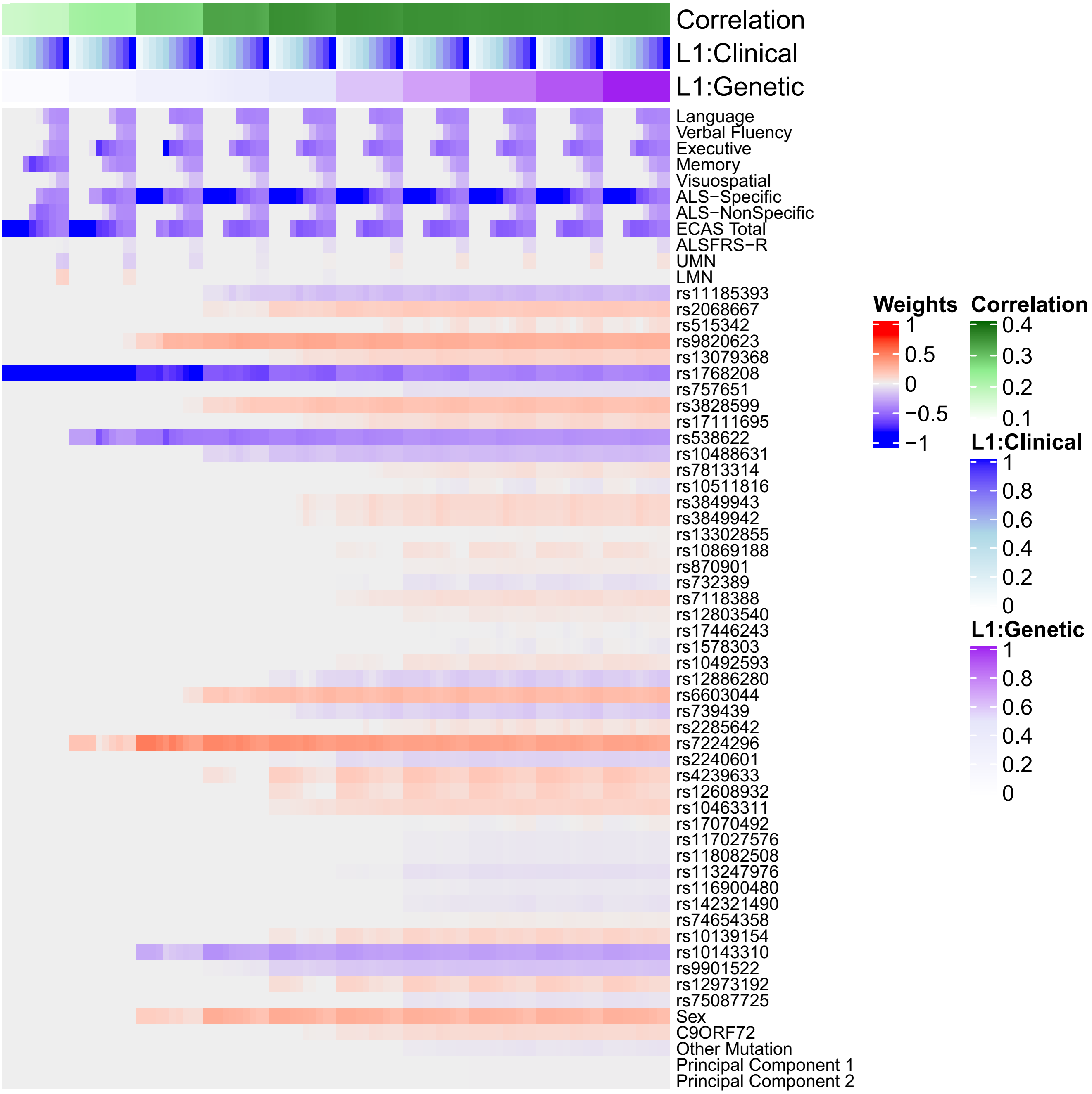

Figure EV1. Grid search for sCCA L1 parameters.

Each column indicates 1 of 100 unique combinations of L1 parameters (ranging 0.1 to 1) applied to clinical and genetic datasets, and each row lists a variable entered into the sCCA. The heatmap denotes the canonical weight strength for each variable; warmer colors indicate positive weights and cooler colors indicate negative weights.

© 2021 The Authors

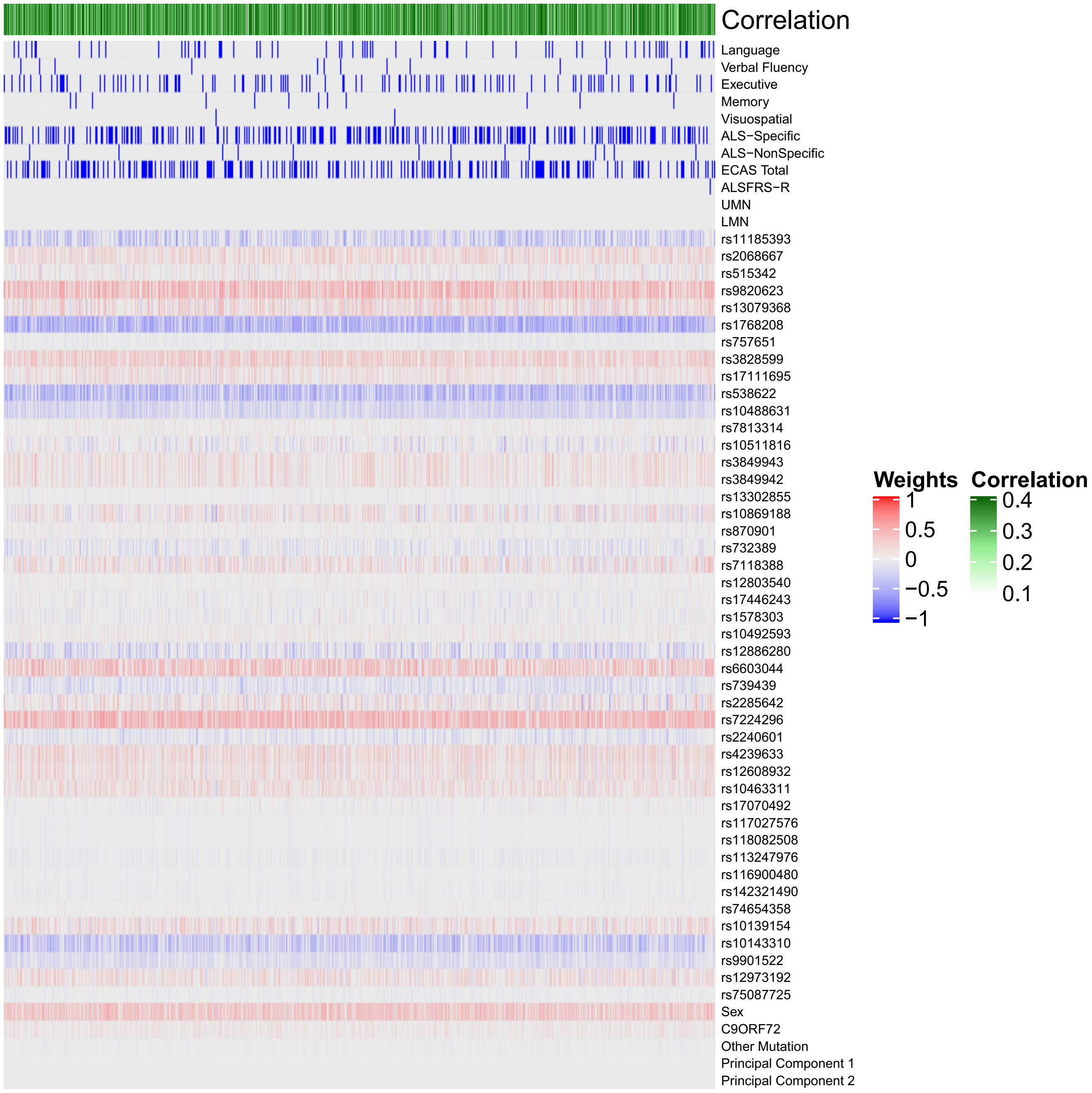

Figure EV2. Bootstrapped sCCA modeling.

Each column indicates 1 of 10,000 iterations of sCCA in each iteration a randomly bootstrapped subsample of 75% of participants in the CReATe PGB cohort was employed. Each row lists a variable entered into the sCCA. The heatmap denotes the canonical weight strength for each variable; warmer colors indicate positive weights and cooler colors indicate negative weights.

© 2021 The Authors

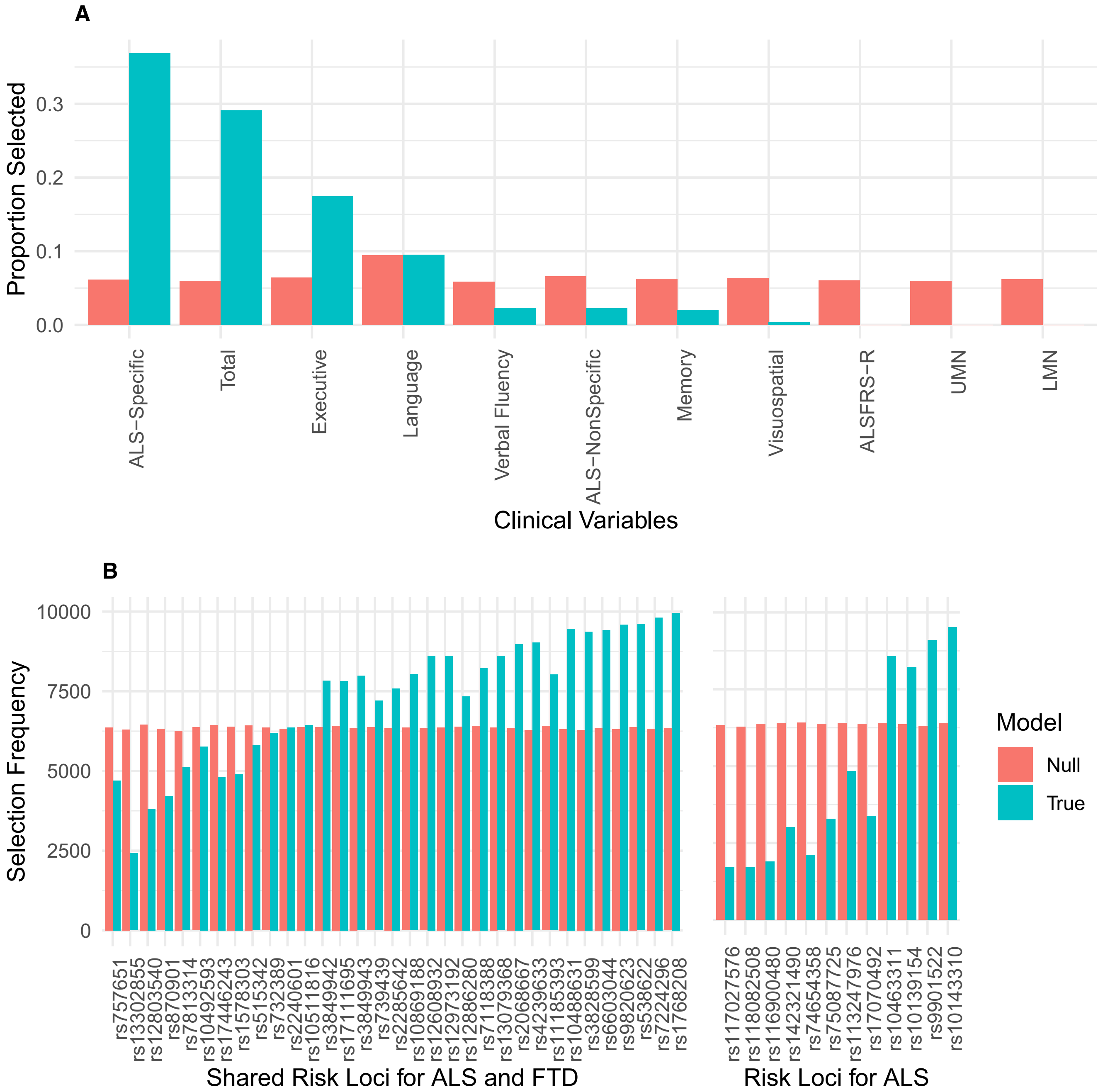

Figure EV3. Variables selected in sCCA modeling.

A Bar graphs demonstrating the proportion of times out of 10,000 iterations that each of the 11 clinical variables were selected by sCCA under true modeling (turquoise) and modeling under the null hypothesis (coral).

B Bar graphs demonstrating the number of times out of 10,000 randomly bootstrapped sCCAs that each of the 45 SNPs were selected by sCCA under true modeling (turquoise) and modeling under the null hypothesis (coral). SNPs are organized according to prior genome-wide association with ALS or joint association with ALS and FTD.

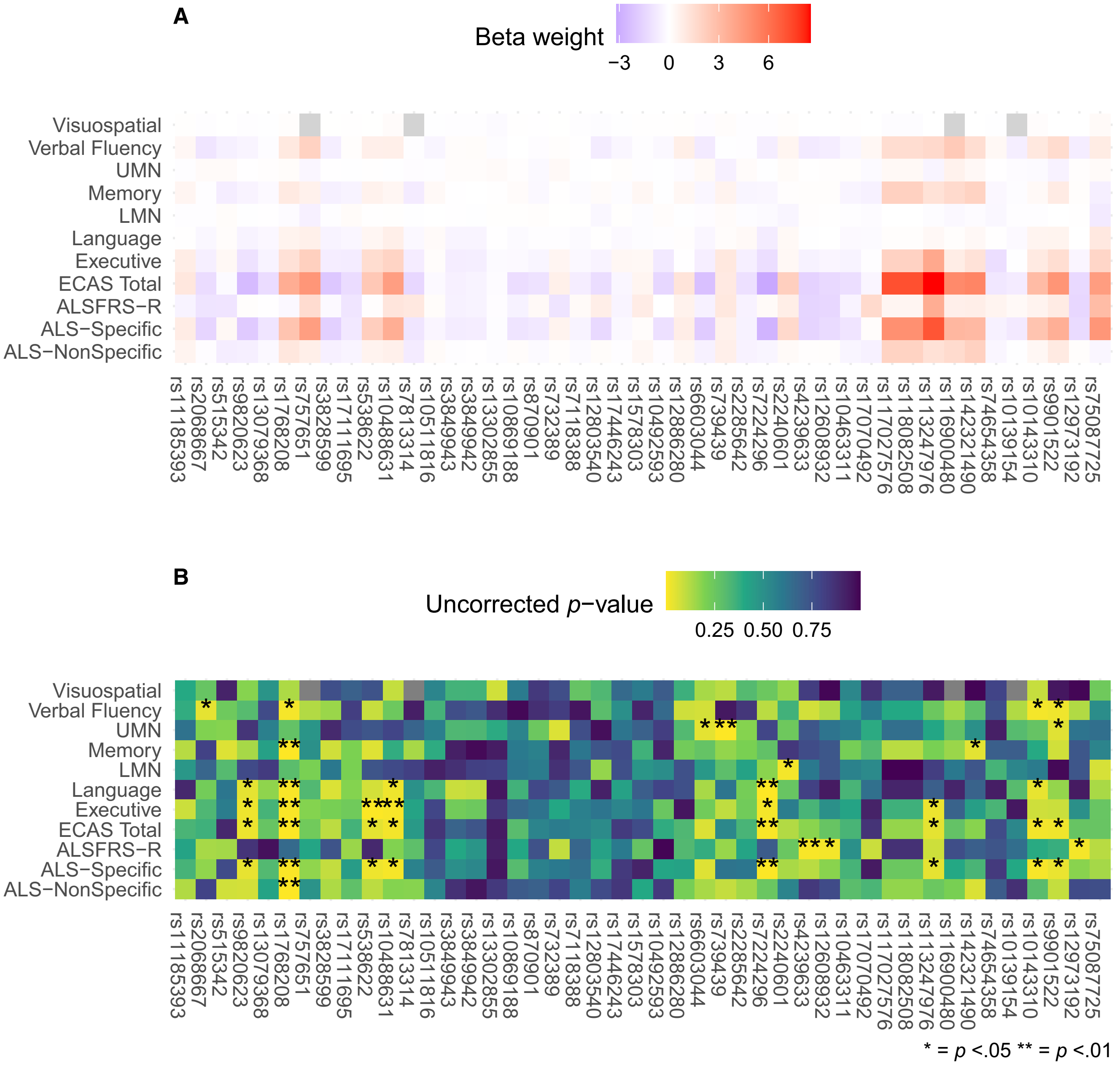

Figure EV4. Univariate SNP associations with clinical variables from linear mixed effects modeling.

A Heatmap of beta weights associated with the fixed effect of each SNP with warmer colors representing positive values and cooler colors representing negative values.

B Heatmap of the *p* value corresponding to the *t* value for the beta weights depicted in A, with brighter colors representing smaller values and darker colors representing larger values. A single star (*) denotes $P < 0.05$, and two stars (**) denote $P < 0.01$.

 © 2021 The Authors

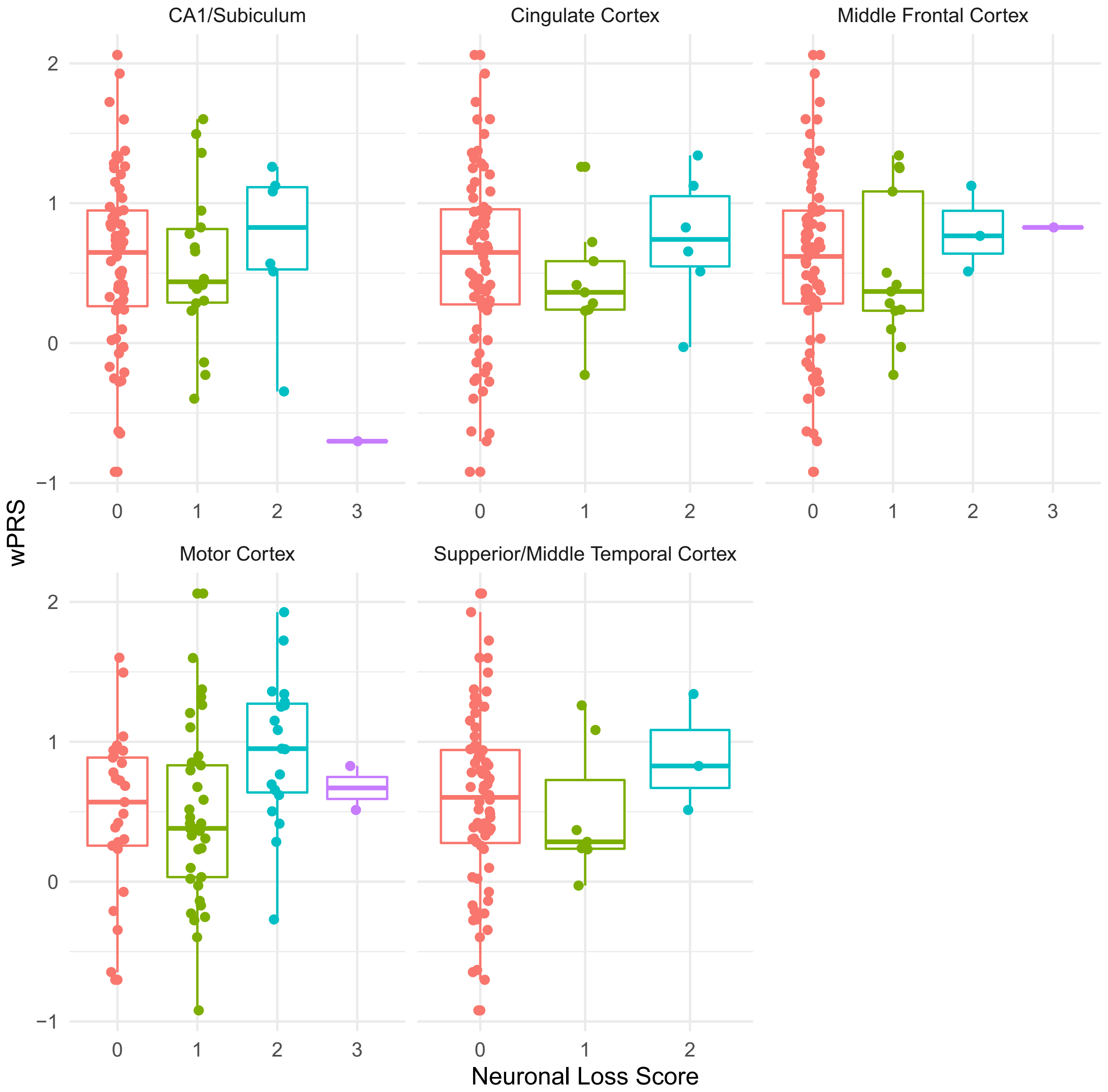

Figure EV5. Magnitude of neuronal loss in ALS cases at autopsy relative to wPRS.

Beeswarm boxplots of ordinal measures of neuronal loss in ALS cases from the UPenn Biobank autopsy cohort relative to wPRS in the cingulate cortex, motor cortex, middle frontal cortex, superior/middle temporal cortex, and hippocampus. The central bands indicate the median, the box indicates the interquartile range (IQR) between the first and third quartiles, and the upper and lower whiskers indicate the largest or smallest value no further than the IQR multiplied by 1.5. For each brain region each data point represents a unique case.

© 2021 The Authors
