# Supplementary material for: Machine learning suggests polygenic risk for cognitive dysfunction in amyotrophic lateral sclerosis
Source: EMBO Mol Med. 2020 Dec 3;13(1):e12595. doi: 10.15252/emmm.202012595 (PMC7799365; doi:10.15252/emmm.202012595)
Supplement: Supplementary file 3 — Table EV1 [file EMMM-13-e12595-s003.docx]

Table EV 1: List of genetic variants analyzed in the CReATe PGB Study.

| **Marker Name** | **Nearest Gene** | **Chr** | **1000 Genome GMAF** | **GRCh38 Position** | **Proxy Marker** | **Proxy HG19.Position** |
| --- | --- | --- | --- | --- | --- | --- |
| rs2068667 | *NFASC* | 1 | 0.208 | chr1:204948552 | rs11240317 | chr1:204920322 |
| rs11185393 | *AMY1A* | 1 | 0.368 | chr1:104209379 | rs67205957 | chr1:104752258 |
| rs515342 | *ASB1* | 2 | 0.214 | chr2:238458655 | rs508986 | chr2:239337691 |
| rs9820623 | *MOBP* | 3 | 0.406 | chr3:39452367 | rs6765697 | chr3:39493239 |
| rs13079368 | *MOBP* | 3 | 0.275 | chr3:39471060 | rs1464047 | chr3:39526874 |
| rs1768208 | *MOBP* | 3 | 0.323 | chr3:39481512 | rs616147 | chr3:39534481 |
| rs10463311 | *TNIP1* | 5 | 0.431 | chr5:151031274 | - | - |
| rs3828599 | *GPX3* | 5 | 0.417 | chr5:151022235 | rs4958872 | chr5:150402334 |
| rs538622 | *ERGIC1* | 5 | 0.32 | chr5:172920676 | rs2446192 | chr5:172352369 |
| rs17111695 | *NAF1* | 5 | 0.183 | chr5:151052885 | rs12518386 | chr5:150438085 |
| rs757651 | *REEP2* | 5 | 0.016 | chr5:138455779 | rs149312547 | chr5:137792021 |
| rs10488631 | *TNPO3* | 7 | 0.059 | chr7:128954129 | rs12539741 | chr7:128596805 |
| rs17070492 | *LOC101927815* | 8 | 0.208 | chr8:2563763 | - | - |
| rs7813314 | *BC045738* | 8 | 0.2 | chr8:2558274 | rs6996532 | chr8:2417678 |
| rs10869188 | *C9ORF72* | 9 | 0.49 | chr9:72614090 | rs7032232 | chr9:75229116 |
| rs870901 | *AK097706* | 9 | 0.133 | chr9:107086201 | rs60743641 | chr9:109854824 |
| rs10511816 | *MOBKL2B* | 9 | 0.206 | chr9:27468463 | rs12551344 | chr9:27466817 |
| rs3849943 | *C9ORF72* | 9 | 0.183 | chr9:27543384 | - | - |
| rs3849942 | *C9ORF72* | 9 | 0.183 | chr9:27543283 | - | - |
| rs13302855 | *C9ORF72* | 9 | 0.086 | chr9:27595997 | rs34460171 | chr9:27594491 |
| rs3849943 | *C9ORF72* | 9 | 0.183 | chr9:27543384 | - | - |
| rs732389 | *AK294518* | 10 | 0.205 | chr10:78584745 | rs7071538 | chr10:80338173 |
| rs7118388 | *CAT* | 11 | 0.454 | chr11:34432600 | rs1962369 | chr11:34456941 |
| rs12803540 | *CAT* | 11 | 0.138 | chr11:34471200 | rs17881488 | chr11:34492443 |
| rs117027576 | *KIF5A* | 12 | 0.00913 | chr12:56922819 | - | - |
| rs113247976 | *KIF5A* | 12 | 0.007 | chr12:57581917 | - | - |
| rs142321490 | *KIF5A* | 12 | 0.006 | chr12:58282349 | - | - |
| rs74654358 | *TBK1* | 12 | 0.012 | chr12:64488187 | - | - |
| rs118082508 | *KIF5A* | 12 | 0.005 | chr12:5692503 | - | - |
| rs116900480 | *KIF5A* | 12 | 0.006 | chr12:58262322 | - | - |
| rs1578303 | *HTR2A* | 13 | 0.204 | chr13:47389011 | rs144877054 | chr13:47962781 |
| rs10492593 | *PCDH9* | 13 | 0.121 | chr13:66919985 | rs73208976 | chr13:67486924 |
| rs17446243 | *TTL/TEL* | 13 | 0.116 | chr13:40174794 | rs78375967 | chr13:40751567 |
| rs10139154 | *SCFD1* | 14 | 0.428 | chr14:30678292 | - | - |
| rs10143310 | *ATXN3* | 14 | 0.339 | chr14:92074037 | - | - |
| rs12886280 | *NUBPL* | 14 | 0.412 | chr14:31829453 | rs35875023 | chr14:32298974 |
| rs6603044 | *BTBD1* | 15 | 0.332 | chr15:83015059 | rs12904695 | chr15:83700365 |
| rs9901522 | *PMP22* | 17 | 0.18 | chr17:14770617 | - | - |
| rs739439 | *KIAA0524* | 17 | 0.105 | chr17:28396803 | rs35714695 | chr17:26719788 |
| rs2240601 | *MSI2* | 17 | 0.192 | chr17:57673751 | rs16942143 | chr17:55748611 |
| rs2285642 | *GGNBP2* | 17 | 0.407 | chr17:36556904 | rs10707226 | chr17:34916453 |
| rs7224296 | *NSF* | 17 | 0.472 | chr17:46722680 | rs9912530 | chr17:44836302 |
| rs12973192 | *UNC13A* | 19 | 0.278 | chr19:17642430 | - | - |
| rs12608932 | *UNC13A* | 19 | 0.43 | chr19:17641880 | rs12973192 | chr19:17753239 |
| rs4239633 | *UNC13A* | 19 | 0.28 | chr19:17631660 | rs71162163 | chr19:17744075 |
| rs75087725 | *C21orf72* | 21 | 0.003 | chr21:44333234 | - | - |

Abbreviations: GMAF = global minor allele frequency; Chr = chromosome; GRCh38 = Genome Reference Consortium Human Build 38; HG19 = Human Genome Project 19
