# Supplementary material for: Machine learning suggests polygenic risk for cognitive dysfunction in amyotrophic lateral sclerosis
Source: EMBO Mol Med. 2020 Dec 3;13(1):e12595. doi: 10.15252/emmm.202012595 (PMC7799365; doi:10.15252/emmm.202012595)
Supplement: Supplementary file 4 — Table EV2 [file EMMM-13-e12595-s004.docx]

Table EV2: Linkage disequilibrium among studied single nucleotide polymorphisms.

| **RSID1** | **RSID2** | **Chr** | **R^2^** | **D'** |
| --- | --- | --- | --- | --- |
| rs11185393 | rs2068667 | 1 | 0.001 | 0.03 |
| rs9820623 | rs13079368 | 3 | 0.657 | 0.816 |
| rs9820623 | rs1768208 | 3 | 0.396 | 0.986 |
| rs13079368 | rs1768208 | 3 | 0.402 | 1 |
| rs3828599 | rs10463311 | 5 | 0.965 | 0.995 |
| rs3828599 | rs17111695 | 5 | 0.031 | 0.237 |
| rs3828599 | rs538622 | 5 | 0 | 0.028 |
| rs10463311 | rs17111695 | 5 | 0.029 | 0.232 |
| rs10463311 | rs538622 | 5 | 0 | 0.027 |
| rs17111695 | rs538622 | 5 | 0 | 0.017 |
| rs3849942 | rs3849943 | 9 | 1 | 1 |
| rs3849942 | rs10869188 | 9 | 0.001 | 0.074 |
| rs10869188 | rs3849943 | 9 | 0.001 | 0.074 |
| rs10139154 | rs12886280 | 14 | 0 | 0.014 |
| rs10139154 | rs10143310 | 14 | 0 | 0.009 |
| rs12886280 | rs10143310 | 14 | 0 | 0.031 |
| rs9901522 | rs739439 | 17 | 0 | 0.032 |
| rs9901522 | rs2285642 | 17 | 0 | 0.031 |
| rs9901522 | rs7224296 | 17 | 0.001 | 0.061 |
| rs9901522 | rs2240601 | 17 | 0.001 | 0.252 |
| rs739439 | rs2285642 | 17 | 0 | 0.039 |
| rs739439 | rs7224296 | 17 | 0 | 0.004 |
| rs739439 | rs2240601 | 17 | 0.002 | 0.176 |
| rs2285642 | rs7224296 | 17 | 0 | 0.01 |
| rs2285642 | rs2240601 | 17 | 0 | 0.01 |
| rs7224296 | rs2240601 | 17 | 0.001 | 0.073 |
| rs4239633 | rs12608932 | 19 | 0.84 | 0.925 |
| rs4239633 | rs12973192 | 19 | 0.836 | 0.92 |
| rs12608932 | rs12973192 | 19 | 0.952 | 0.991 |

Abbreviations: RSID = reference single nucleotide polymorphism (SNP) identification number; RSID2 = reference SNP identification number 2; Chr = chromosome
