# Supplementary material for: Machine learning suggests polygenic risk for cognitive dysfunction in amyotrophic lateral sclerosis
Source: EMBO Mol Med. 2020 Dec 3;13(1):e12595. doi: 10.15252/emmm.202012595 (PMC7799365; doi:10.15252/emmm.202012595)
Supplement: Supplementary file 5 — Table EV3 [file EMMM-13-e12595-s005.docx]

Table EV3: Peak voxel coordinates for regions of reduced cortical thickness in ALS patients relative to healthy controls, and peak voxel coordinates for regions of reduced cortical thickness associated with higher weighted polygenic score (wPRS) in patients with ALS from the UPenn Biobank neuroimaging cohort.

| Neuroanatomic region (BA) | | **L/R** | **MNI Coordinates**  **x y z** | | | ***T* statistic** | ***p* value** | **Voxels** |
| --- | --- | --- | --- | --- | --- | --- | --- | --- |
| *Reduced cortical thickness in ALS relative to healthy controls*^1^*:* | | | | | | | | |
|  | |  |  |  |  |  | <.001 | 56969 |
| Orbitofrontal cortex (11) | | R | 10 | 26 | -24 | 6.41 |  |  |
| Insula (13) | | R | 34 | 16 | -18 | 6.18 |  |  |
| Insula (13) | | L | -32 | 26 | 2 | 6.12 |  |  |
| Inferior frontal gyrus (45) | | R | 36 | 22 | 6 | 6.06 |  |  |
| Anterior cingulate cortex (32) | | - | 0 | 46 | 8 | 5.68 |  |  |
| Insula (13) | | L | -40 | 16 | -4 | 5.59 |  |  |
| Dorsolateral prefrontal cortex (9) | | L | -2 | 46 | 18 | 5.36 |  |  |
| Insula (13) | | R | 42 | 4 | 2 | 5.32 |  |  |
| Insula (13) | | L | -30 | 16 | 8 | 5.29 |  |  |
| Anterior prefrontal cortex (10) | | R | 24 | 56 | -4 | 5.18 |  |  |
| *Reduced cortical thickness associated with wPRS in ALS:* | | | | | | | | |
| Anterior premotor cortex (8) | L | | -34 | 12 | 36 | 3.99 | <.0001 | 112 |
| Parahippocampal gyrus (36) | R | | 30 | -40 | -4 | 3.22 | .001 | 111 |
| Hippocampus (54) | L | | -24 | -32 | -6 | 2.91 | .002 | 109 |
| Orbital frontal cortex (47) | R | | 34 | 40 | -8 | 3.01 | .002 | 108 |
| Anterior cingulate cortex (32) | R | | 12 | 42 | 0 | 3.06 | .002 | 61 |
| Dorsolateral prefrontal cortex (9) | R | | 34 | 18 | 24 | 3.51 | <.0001 | 57 |
| Fusiform Gyrus (37) | R | | 56 | -50 | 10 | 2.91 | .002 | 56 |
| Anterior prefrontal cortex (10) | L | | -4 | 54 | 12 | 2.93 | .002 | 43 |
| Orbitofrontal cortex (11) | R | | 10 | 44 | -20 | 2.83 | .002 | 40 |
| Superior parietal cortex (40) | R | | 56 | -26 | 24 | 2.95 | .002 | 37 |
| Anterior prefrontal cortex (10) | L | | -32 | 52 | 6 | 2.94 | .002 | 31 |
| Orbital frontal cortex (47) | R | | 46 | 34 | -6 | 2.75 | .002 | 30 |
| Lateral temporal cortex (21) | L | | -64 | -44 | 4 | 2.69 | .004 | 29 |
| Anterior temporal cortex (38) | R | | 46 | 8 | -28 | 2.76 | .003 | 26 |
| Anterior prefrontal cortex (10) | R | | 22 | 56 | -6 | 2.55 | .006 | 25 |
| Orbitofrontal cortex (11) | R | | 12 | 66 | -20 | 3.24 | .001 | 23 |
| Fusiform Gyrus (37) | R | | 58 | -50 | -2 | 2.62 | .005 | 17 |
| Orbital frontal cortex (47) | R | | 42 | 44 | -8 | 2.66 | .004 | 16 |
| Anterior cingulate cortex (32) | L | | -4 | 22 | 34 | 2.52 | .007 | 15 |
| Dorsolateral prefrontal cortex (9) | R | | 48 | 26 | 22 | 2.56 | .006 | 14 |
| Orbital frontal cortex (47) | L | | -44 | 36 | -6 | 2.89 | .002 | 12 |

Abbreviations: BA = Brodmann area, L/R = Left/Right, MNI = Montreal Neurological Institute. Note.^1^ Cortical regions identified from peak voxel coordinates in an effort to describe sub-peaks within a larger, contiguous cluster.
