# Supplementary material for: Machine learning suggests polygenic risk for cognitive dysfunction in amyotrophic lateral sclerosis
Source: EMBO Mol Med. 2020 Dec 3;13(1):e12595. doi: 10.15252/emmm.202012595 (PMC7799365; doi:10.15252/emmm.202012595)
Supplement: Supplementary file 6 — Table EV4 [file EMMM-13-e12595-s006.docx]

Table EV4: Number of UPenn Biobank ALS autopsy cases for each neuropathological measurement in each sampled neuroanatomical region.

| **Region** | **Neuropathological Measurement** | **N** |
| --- | --- | --- |
| Middle frontal cortex | Neuronal loss | 86 |
| Middle frontal cortex | TDP-43 | 86 |
| Cingulate cortex | Neuronal loss | 87 |
| Cingulate cortex | TDP-43 | 86 |
| Motor cortex | Neuronal loss | 83 |
| Motor cortex | TDP-43 | 85 |
| Superior / middle temporal cortex | Neuronal loss | 86 |
| Superior / middle temporal cortex | TDP-43 | 83 |
| CA1 / subiculum (hippocampus) | Neuronal loss | 87 |
| CA1 / subiculum (hippocampus) | TDP-43 | 84 |

Abbreviations: CA1 = cornu ammonis 1; TDP-43 = TAR DNA-binding protein [43 kDa]
